# MixtureTree: a program for constructing phylogeny

**DOI:** 10.1186/1471-2105-12-111

**Published:** 2011-04-21

**Authors:** Shu-Chuan Chen, Michael S Rosenberg, Bruce G Lindsay

**Affiliations:** 1 School of Mathematical and Statistical Sciences, Arizona State University, Tempe, AZ 85287, USA; 2 Center for Evolutionary Medicine and Informatics, Biodesign Institute, and School of Life Sciences, Arizona State University, Tempe, AZ 85287, USA; 3 Department of Statistics, Pennsylvania State University, University Park, PA 16802, USA

## Abstract

**Background:**

MixtureTree v1.0 is a Linux based program (written in C++) which implements an algorithm based on mixture models for reconstructing phylogeny from binary sequence data, such as single-nucleotide polymorphisms (SNPs). In addition to the mixture algorithm with three different optimization options, the program also implements a bootstrap procedure with majority-rule consensus.

**Results:**

The MixtureTree program written in C++ is a Linux based package. The User's Guide and source codes will be available at http://math.asu.edu/~scchen/MixtureTree.html

**Conclusions:**

The efficiency of the mixture algorithm is relatively higher than some classical methods, such as Neighbor-Joining method, Maximum Parsimony method and Maximum Likelihood method. The shortcoming of the mixture tree algorithms, for example timing consuming, can be improved by implementing other revised Expectation-Maximization(EM) algorithms instead of the traditional EM algorithm.

## Background

Methods for constructing a phylogeny given a set of the DNA sequences is always a popular topic in both biological and statistical research. Many classical methods are popular, such as Neighbor-Joining (NJ) method, Maximum Parsimony (MP) method, Maximum Likelihood (ML) method, and Bayesian (MCMC) approaches ([1] and [2]). There are also many programs which implement these methods, including PHYLIP [3], PAUP [4], and MEGA [5]. Chen and Lindsay introduced a mixture likelihood algorithm as a novel and natural way to deal with such problems because the distribution of offsprings' sequence is *a mixture *of parental distributions [6]. Unlike the classic methods, this approach uses the frequencies of each sequence within the population to help reconstructing the phylogeny from binary sequences. The model proposed by Chen and Lindsay [6] was done mathematically, as a first step, and that it should not be particularly problematic with most SNPs are bi-allelic with only a very small proportion (well less than 1% and probably less than 0.1%) tri-allelic or quad-allelic ([7] and [8]). The algorithm uses a K-component bernoulli mixture to model binary sequences. It is well-known that when the parameter takes values in an infinite dimensional space, the maximum likelihood estimation often fails. To overcome the above issue, we can first maximize over a constrained subspace of the parameter space then relax the constraint as the sample size grows. In this case, the maximum likelihood estimation will then work. The parameter used to create a constrained subspace is called a sieve parameter [9]. In our case, the sieve parameter is the mutation rate *p *which is considered as a function of time in the mixture model. By varying *p *from 0 to 0.5, the mixture algorithm can give an estimate of the recent common ancestors of the given sequences. In order to obtain the mixture tree of the observed sequences, the Expectation-Maximization (EM) algorithm is employed. To overcome the small weights (*π*) problem in the regular EM, the nature way comes up is that we do not update the weights *π*. Such an algorithm, we call it FixEM. The Modal EM is a nonparametric statistical approach to clustering via mode identification in the Bernoulli mixtures ([10] and [11]). The MixtureTree program contains the regular EM algorithm plus these two related optimization algorithms, Fixed EM (FixEM) algorithm and Modal EM (ModalEM) algorithm. Any and all can be chosen to estimate the ancestral sequences. We have found that the FixEM and ModalEM algorithms have better computational efficiency over the regular EM algorithm [11]. After constructing the phylogeny, it is common for researchers to carry out a nonparametric bootstrap analysis ([12,13], and [14]) in which one creates bootstrap samples from the empirical distribution of sites from the original sequence data. The MixtureTree algorithm also implements a majority-rule consensus tree method from PHYLIP. This method is originally proposed by Margush and McMorris [15] and also allows one to easily find the strict consensus tree.

## Implementation

The input function in the MixtureTree program can read DNA sequences in a simple tabular format, in which all the sequences should be stacked in the form:

*Sequence Name*   *Sequence*   *Sequence' s Frequency*

The parameters setting can be changed in the parameter file. Three different optimization options can be chosen in the algorithm. The output function of the program writes the estimated mixture tree in the commonly-used Newick format which can be read, viewed, and manipulated by many other programs. Whether the EM algorithm converges can be checked in the output file *em.txt*. If chosen as an option, the bootstrap trees will also be output in Newick format. The summary of the bootstrap trees will be in a separate le. All of the output trees can be easily imported into other packages, such as the R package APE for further manipulations. Details can be found in the UserGuide at http://math.asu.edu/~scchen/MixtureTree.html.

## Results and Discussion

**Figure 1:**
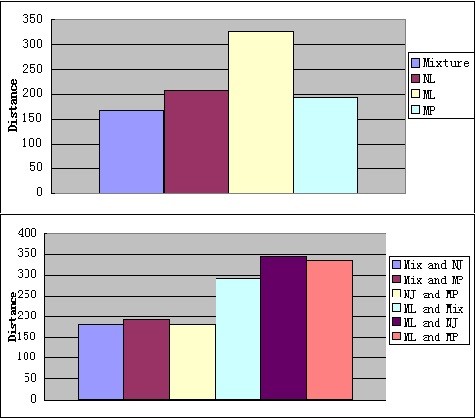
**Results of comparisons when mutation rate is 0.00000375, length of the sequence is 10, the number of lineages is 5 and the sample size is 200**.

In order to evaluate the efficiency of the MixtureTree algorithm, we generated a sample of size 200 by using the simulator ms ([16]) with five lineages in each sample unit. The simulator generates the true phylogeny along with the lineages, so we reconstruct the mixture tree, Neighbor-Joining tree and Maximum Parsimony tree based on the simulated lineages and then compare them with the true tree by using the Robinson and Foulds metric ([17]). The metric proposed by Robinson and Foulds ([17]) is based on elementary operations on transforming one tree to another tree in order to compare two tree topologies. By comparing the distances of trees, we can make conclusion of tree A is closer to tree B rather than to tree C. For each simulation we also calculate the distances among the Mixture tree, Neighbor-Joining tree, Maximum Parsimony tree and Maximum Likelihood tree then we sum over the distances from those 200 simulations. In the simulation study, we simulated 200 sets of sequences using various mutation rates. For each set of sequences the length of the sequences was 10 SNPs and the number of lineages was 5. With small mutation rates, we received very similar results. For example, with mutation rate 0.00000375 the sum of the distances of these 200 simulations for MixtureTree, NJ tree, MP tree, and ML tree are 168, 208, 194 and 328. The three methods, except ML tree, were about as different from each other as they were from the true tree, showing that the new mixture tree method is fundamentally different from the other two. The sum of the pairwise distances of Mixture v.s. NJ, Mixture v.s. MP, NJ v.s. MP, Mixture v.s. ML, NJ v.s. ML, MP v.s. ML are 180, 192, 180, 292, 346, 334 and shows that the performance of MixtureTree is closer to NJ tree. The results are presented using histograms in Figure 1.

## Conclusions

From the comparison above, we can see that the efficiency of the mixture algorithm is relatively higher than the other three methods. However, there are also some shortcomings of the mixture algorithm, for example, it is more time-consuming to obtain the phylogeny than the other two methods. This shortcoming can be solved by implementing the Fixed EM or Modal EM instead of the traditional EM algorithm.

## Availability and requirements

The MixtureTree construction project and source codes can be found in the link http://math.asu.edu/~scchen/MixtureTree.html. It is Linux based program, written in C++, which implements an algorithm based on mixture models for reconstructing phylogeny from binary sequence data, such as single-nucleotide polymorphisms (SNPs). Any user uses the program needs to cite the MixtureTree website and the papers listed there.

## Authors' contributions

SC implemented the computational model, carried out the simulations and drafted the manuscript. MR implemented the computational model and biological interpretation. BL participated in summarizing and interpretation the simulation results and revising the manuscript. All authors read and approved the final manuscript.

## References

[B1] Huelsenbeck JP Ronquist F Nielsen R Bollback JP Bayesian inference of phylogeny and its impact on evolutionary biology Science 2001 294 2310 2314 10.1126/science.1065889 11743192

[B2] Ronquist F Huelsenbeck JP MRBAYES 3: Bayesian phylogenetic inference under mixed models Bioinformatics 2003 19 1572 1574 10.1093/bioinformatics/btg180 12912839

[B3] Felsenstein J PHYLIP - Phylogeny Inference Package (Version 3.2) Cladistics 1989 5 164 166

[B4] Swofford DL PAUP*. Phylogenetic Analysis Using Parsimony (*and Other Methods) 1998 Sunderland: MA: Sinauer

[B5] Kumar S Dudley J Nei M Tamura K MEGA: A biologist-centric software for evolutionary analysis of DNA and protein sequences Briefings in Bioinformatics 2008 9 299 306 10.1093/bib/bbn017 18417537 PMC2562624

[B6] Chen SC Lindsay B Building mixture trees from binary sequence data Biometrika 2006 93 4 843 860 10.1093/biomet/93.4.843

[B7] Ohtani T Ikeda S Lwin H Arai T Muramatsu M Sawabe M Polymorphisms of the formylpeptide receptor gene (FPR1) and susceptibility to stomach cancer in 1531 consecutive autopsy cases Biochemical and Biophysical Research Communications in press 10.1016/j.bbrc.2010.12.136 21216225

[B8] Harismendy O Bansal V Bhatia G Nakano M Scott M Wang X Dib C Turlotte E Sipe JC Murray SS Deleuze JF Bafna V Topol J E Frazer KA Population sequencing of two endocannabinoid metabolic genes identifies rare and common regulatory variants associated with extreme obesity and metabolite level Genome Biology 2010 11 11 R118 10.1186/gb-2010-11-11-r118 21118518 PMC3156957

[B9] Geman S Hwang CR Nonparametric maximum likelihood estimation by the method of sieves The Annals of Statistics 1982 10 2 401 414 10.1214/aos/1176345782

[B10] Li J Ray S Lindsay B A nonparametric statistical approach to clustering via mode identification Journal of Machine Learning Research 2007 8 1687 1723

[B11] Chen SC Li M Rosenberg M Lindsay B Lu HS, Schölkopf B, Zhao H Mixture tree construction and its applications Handbook of Computational Statistics: Statistical Bioinformatics Springer-Verlag in press

[B12] Felsenstein J Statistical inference of phylogenies (with Discussion) J R Statist Soc A 1983 146 246 272 10.2307/2981654

[B13] Felsenstein J Inferring Phylogenies 2003 Boston: Sinauer

[B14] Holmes S Bootstrapping phylogenetic trees: theory and methods Statist Sci 2003 18 241 255 10.1214/ss/1063994979

[B15] Margush T McMorris FR Note Consensus n-Trees Bulletin of Mathematical Biology 1981 43 2 239 244

[B16] Hudson RR Generating samples under a Wright-Fisher neutral model of genetic variation Bioinformatics 2002 18 337 338 10.1093/bioinformatics/18.2.337 11847089

[B17] Robinson DF Foulds LR Comparison of Phylogenetic Trees Mathematical Biosciences 1981 53 131 147 10.1016/0025-5564(81)90043-2

